# New Insights into Ferroptosis Initiating Therapies (FIT) by Targeting the Rewired Lipid Metabolism in Ovarian Cancer Peritoneal Metastases

**DOI:** 10.3390/ijms232315263

**Published:** 2022-12-03

**Authors:** Shijie Zhan, Mingo M. H. Yung, Michelle K. Y. Siu, Peili Jiao, Hextan Y. S. Ngan, David W. Chan, Karen K. L. Chan

**Affiliations:** 1 Department of Obstetrics & Gynaecology, Li Ka Shing Faculty of Medicine, The University of Hong Kong, Hong Kong SAR, China; 2 School of Medicine, The Chinese University of Hong Kong-Shenzhen, Shenzhen 518172, China

**Keywords:** ferroptosis, fatty acid metabolism (FAM), tumor microenvironment (TME), peritoneal metastasis, chemoresistance, ascites, omentum, ovarian cancer

## Abstract

Ovarian cancer is one of the most lethal gynecological cancers worldwide. The poor prognosis of this malignancy is substantially attributed to the inadequate symptomatic biomarkers for early diagnosis and effective remedies to cure the disease against chemoresistance and metastasis. Ovarian cancer metastasis is often relatively passive, and the single clusters of ovarian cancer cells detached from the primary ovarian tumor are transcoelomic spread by the peritoneal fluid throughout the peritoneum cavity and omentum. Our earlier studies revealed that lipid-enriched ascitic/omental microenvironment enforced metastatic ovarian cancer cells to undertake metabolic reprogramming and utilize free fatty acids as the main energy source for tumor progression and aggression. Intriguingly, cell susceptibility to ferroptosis has been tightly correlated with the dysregulated fatty acid metabolism (FAM), and enhanced iron uptake as the prominent features of ferroptosis are attributed to the strengthened lipid peroxidation and aberrant iron accumulation, suggesting that ferroptosis induction is a targetable vulnerability to prevent cancer metastasis. Therefore, the standpoints about tackling altered FAM in combination with ferroptosis initiation as a dual-targeted therapy against advanced ovarian cancer were highlighted herein. Furthermore, a discussion on the prospect and challenge of inducing ferroptosis as an innovative therapeutic approach for reversing remedial resistance in cancer interventions was included. It is hoped this proof-of-concept review will indicate appropriate directions for speeding up the translational application of ferroptosis-inducing compounds (FINs) to improve the efficacy of ovarian cancer treatment.

## 1. Introduction—An Overview of the Prevalence and Burden of Ovarian Cancer 

Ovarian cancer is the second most common type of gynecological cancer. Being the seventh most diagnosed malignancy around the globe, it contributes to around 239,000 new cancer cases and leads to 152,000 deaths annually [1]. It is estimated that 1.28% of women are diagnosed with ovarian cancer during their lifetime [2]. Both perimenopausal and postmenopausal women can suffer from ovarian cancer, possessing more than 80% of cases diagnosed in individuals over 40 years old and peaking the incidence rate at 60 [3]. The risk of ovarian cancer seemingly increases with age, having more than 50% of the diagnoses occurring in women over the age of 60 [2]. However, the incidence rate of ovarian cancer varied greatly between races and ethnicities that Caucasian women were more commonly diagnosed with the malignancy than non-Hispanic black and Asian women [2,4]. Nonetheless, the greatest mortality rate is observed in African women, as they may have limited access to therapeutic and diagnostic resources. Ovarian cancer is so prevalent that the estimated number of new cases worldwide shall exceed 276,000 annually by 2030 [2]. 

In Hong Kong, ovarian cancer is the sixth most common cancer among women, accounting for approximately 3.9% of malignancy cases and 4% of cancer-related deaths, respectively [5]. In 2019, more than 700 individuals were diagnosed with ovarian cancer, and the indigenous incidence rate had shown a steady increase of 1.4% annually [5]. Although a significant reduction in mortality rate had been observed in patients with stage II ovarian cancer, the mortality rate did not show a remarkable change over the decades. As indicated by more than 70% of patients being diagnosed at advanced stages accompanied by peritoneal metastases [6], they usually have a poor chance of treatment success and a high risk of aggressive tumor recurrence within 1.5 years from diagnosis as a result of the emergence of acquired chemoresistance and minimal residual disease (MRD), having an abysmal five-year survival rate less than 40% [7,8,9]. 

## 2. Difficulties in Current Ovarian Cancer Treatment 

With more than 10 histologic subtypes of ovarian cancer identified, they generally originate from three anatomical structures: ovaries, fallopian tubes, or peritoneum [10]. The malignancies are further characterized by the tissues that they originate from, which include epithelial carcinoma, germ cell carcinoma, and stromal cell carcinoma. Among all the subcategories, epithelial ovarian cancer (EOC) is the most commonly diagnosed type, accounting for approximately 90% of ovarian cancer cases [11,12], with high-grade serous ovarian carcinoma (HGSOC) being the most predominant histotype of EOC. The treatment options for ovarian cancer depend on the histological subtypes and stages of the disease. Individual factors of the patient also need to be considered. Accurate staging is critical in cases where the malignancy is confined to one or both ovaries, as the therapeutic regimes are often based on the resulting tumor staging. Currently, research on ovarian cancer is mainly focused on the inadequacies associated with screening and diagnostic methods in the early stages of EOC as well as treatment strategy selection [13], but many trials are unsuccessful in finding novel methods and promising biomarkers for effective detection and prediction of the disease prognosis and therapeutic response as transcriptional heterogeneity is highly observed between and within ovarian cancers of the same histotype [14]. 

Primary cytoreductive surgery (PCS) followed by a combination of platinum-taxane-based neoadjuvant chemotherapy (NACT) remains the standard treatment for advanced ovarian cancer. Yet, the efficacy and long-term side effects are variable [15]. An exploratory analysis illustrated that around one-third of ovarian cancer patients (*n* = 3126) with complete resection exhibited either a small residual tumor burden (1–10 mm in diameter) or macroscopic residual disease (>10 mm in diameter) [16,17]. These substantially hamper the remedial possibility of the disease, leading to a significant plunge in overall survival when the lesions have invaded the pelvic cavity [18]. Metastatic ovarian cancer is believed to have a self-renewing subpopulation of cancer stem cells (CSCs) that usually are inert to cytotoxic chemotherapy, and recurrent chemoresistant ovarian cancer is highly incurable [17,19,20]. Moreover, transcoelomic metastases of advanced ovarian cancer are often associated with the cause of malignant ascites, malnutrition, and cachexia, resulting in metabolic changes and bowel obstruction in patients with ovarian carcinoma [17,21,22,23]. Metastatic ovarian cancer cells detached from the primary lesion and suspended in the malignant ascites underwent a metabolic shift from glycolysis to fatty acid metabolism (FAM), affording their survival and proliferation within the omental tissues as a significant secondary niche [24]. Therefore, elucidating the molecular events underlying therapeutic resistance and metastatic process and unraveling the alternative therapeutic interventions for EOC is a priority in ovarian cancer research and treatment. 

## 3. Alternative Therapeutic Approaches to Ovarian Cancer 

With the limited success of the mentioned therapies on ovarian cancer patients, especially those with late-stage diseases, a plethora of alternative therapeutic strategies have been developed over the decade to improve overall prognosis and survival rates. These alternative options may be performed in line with the traditional primary treatments in a complementary manner instead of a standalone regimen. Accumulating clinical evidence supports the effectiveness of such promising alternative approaches, especially targeted therapy and immunotherapy. 

### 3.1. Targeted Therapy in Ovarian Cancer 

**Figure 1:**
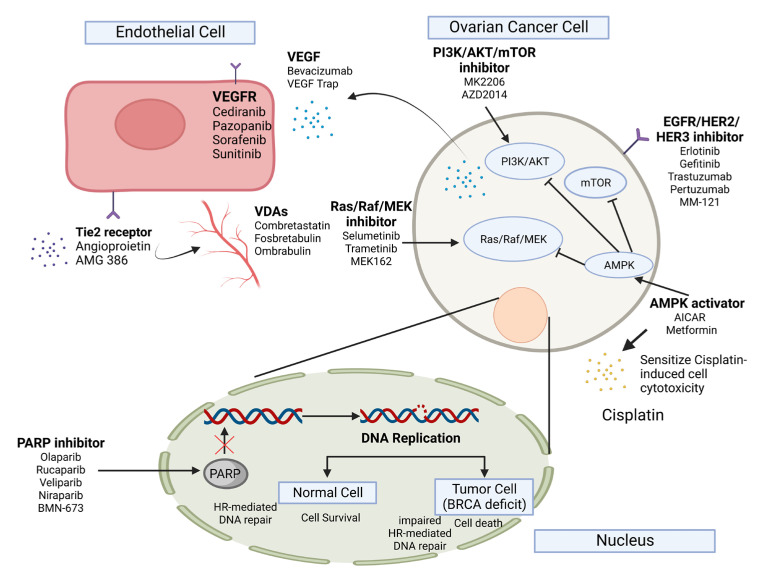
Common targeted therapy in ovarian cancer. Clinical trials of Bevacizumab, a monoclonal antibody against vascular endothelial growth factor A (VEGF-A), have indicated significant efficacy against ovarian cancer and have been proposed to use as a first-line therapy with chemotherapy. Aflibercept is another agent that inhibits vascular endothelial growth factor (VEGF) directly. Tyrosine kinase inhibitors (TKI) that target VEGF receptors and inhibit other angiogenic molecules, including Nintedanib, Pazopanib and Cabozantinib, are currently under investigation in clinical trials. Besides, poly ADP ribose polymerase (PARP) inhibitors may eradicate tumor cells selectively while limiting toxicity to normal cells through synthetic lethality in ovarian cancer resulting from genetic mutations like breast cancer gene 1/2 (BRCA1/2). Furthermore, the RAS/RAF/MEK pathway may serve as an important target for low-grade serous ovarian carcinoma as patients with such disease show a high rate of B-Raf proto-oncogene serine/threonine kinase (BRAF) and Kirsten rat sarcoma virus (KRAS) mutation. Several therapeutic agents for ovarian cancer that modulate the PI3K/AKT/mTOR signaling are under exploration in clinical trials as activation of the pathway is observed frequently in clear cell and endometrioid ovarian cancers. Similarly, activation of AMP-activated protein kinase (AMPK) has been considered to play an important role in suppressing malignancies including ovarian cancer by downregulating a number of pathways such as mTOR/p70S6K and AKT/ERK transductions, making AMPK a potential druggable target for human cancers [38].

The main mechanism of targeted therapy is to inhibit the growth and metastasis of cancer cells by blocking or interfering with specific molecules involved in oncogenesis, therefore minimizing their impact on normal mitotic cells. Through this targeting strategy, the drug per se can elicit an apoptotic response in the cancer cells or assist the host immune system in identifying and eradicating the malignant cells [25]. The most typical types of targeted therapy are either small molecules or monoclonal antibodies. As illustrated in Figure 1, the small molecule targeted therapy agents are frequently able to penetrate the cell membrane of malignant cells and target components within the cell, whereas monoclonal antibody variants often interact with extracellular or cellular surface targets due to their relatively larger molecular size [26,27]. 

One of the major classes of small molecule-targeted therapies for ovarian cancer would be the poly ADP ribose polymerase (PARP) inhibitors (Figure 1). PARP is a protein family involved in the repairing mechanism in single-strand breaks in DNA. By inhibiting the action of PARP in hereditary breast and ovarian cancer (HBOC), gene mutations accumulate and cannot be effectively repaired, ultimately resulting in cell death in the malignant cells [28]. Apoptosis is preferentially induced in PARP-treated cancer cells, as normal cells lacking the genetic mutations of *BRCA1* and *BRCA2* can still function in homologous DNA repair [29]. On the other hand, vascular endothelial growth factor (VEGF), a critical mediator of angiogenesis, was observed to be highly expressed in several ovarian surface epithelial neoplasms, inferring that VEGF-targeting monoclonal antibodies may be a kind of potential anti-angiogenic therapy for the treatment of ovarian cancer (Figure 1). Many therapeutic agents targeting this angiogenesis promoter, such as Avastin^®^ (Bevacizumab), have been applied in specific clinical trials. Some of them exhibited a favorable response rate against ovarian cancer, while several of them displayed moderate efficacy [9,30]. It has been speculated that the inhibited VEGF-mediated signaling may be compensated by other pathways, such as fibroblast growth factors and epidermal growth factors [31]. 

Abovementioned the chemotherapeutic outcomes of most cytotoxic agents are grounded in the ability to elicit an apoptotic response in tumor cells upon drug treatment. This response is further regulated by the effect of tumor suppressors and the modulatory characteristics of cell survival factors [32]. Several gene family groups have been identified to be involved in the anti-apoptotic mechanism and upregulated to sustain the intensive growth of malignant cells, including *PI3K* and *AKT* [33]. Therefore, deregulation in these pathways may result in the failed inducement of apoptosis by the chemotherapeutic drugs, resulting in chemoresistance in the cancer cells. Targeting these augmented oncogenic signalings would be a potential therapeutic strategy in combating human cancers. For example, drugs targeting MEK1/2 and ERK1/2, such as Selumetinib, Cobimetinib, LY3214996 and Trametinib have been shown to sensitize Cisplatin treatment in non-small cell lung cancer [34]. Consistently, our previous investigations demonstrated that AMPK activators such as AICAR and Metformin inhibited both mTOR/p70S6K and AKT/ERK/FOXM1 signals in ovarian cancer cells and produced a synergistic effect on Cisplatin-induced cell cytotoxicity in ovarian cancer cells [35,36,37], suggesting that targeted therapies exert growth inhibition effect on ovarian cancer cells and improve the efficacy of Cisplatin-based chemotherapy in advanced ovarian cancer. 

### 3.2. Immunotherapy in Ovarian Cancer 

Immunotherapy, a type of biological therapy, aims to treat cancer by activating the immune system, while treatment of other diseases, especially autoimmune diseases, may be based on the suppression of it. Numerous studies have shown a significant increase in progression-free and disease-free periods in cancer patients upon immunotherapy [39]. The efficacy of immunotherapy can be further elevated with chemotherapy and radiotherapy. Immunotherapy for ovarian cancer focuses on the presentation of neo-antigens on dendritic cells derived from ovarian cancer cells. Presentation is mediated through MHC class I T-cell receptors and the co-stimulation of CD80. 

**Figure 2:**
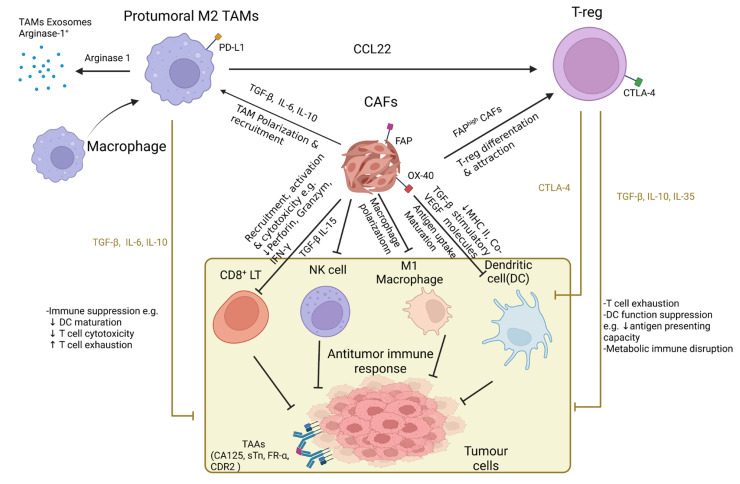
The immunosuppressive microenvironment of ovarian cancer. The ovarian cancer tumor microenvironment (TME) consists of immune cells, including cytotoxic CD8^+^ T lymphocytes (CD8^+^ LT), natural killer cells (NK cells), M1 macrophages and dendritic cells (DC) as well as immune-tolerant cells such as tumor-associated macrophages (TAMs), cancer-associated fibroblasts (CAFs) and regulatory T cells (T-regs). Molecules that inhibited the responses of antitumor immunity, such as cell surface receptors, cytokines and chemokines, were expressed by T-regs, CAFs and TAMs. The presence of cytokines such as Transforming Growth Factor Beta (TGF-β), Interleukin 6 (IL-6), Interleukin 10 (IL-10), Interleukin 35 (IL-35), etc. inhibited CD8^+^ LT recruitment, activation and cytotoxicity, while promoting CD8^+^ LT exhaustion and disrupting DC maturation. CAFs further impeded the antigen presentation functioning of DC through the downregulation of major histocompatibility complex II (MHC II) expression and co-stimulatory molecules on DC by TGF-β. TGF-β also negatively regulated the activation of Natural Killer cells (NK cells) and cytotoxic activity. Production of the cytokine CC motif chemokine ligand 22 (CCL22) by TAMs formed a gradient of chemokines that promoted the accumulation of T-regs in the TME [42].

The potential therapeutic benefits of immunotherapy in ovarian cancer are based on the rising evidence indicating ovarian cancers are immunogenic cancers. Several tumor-associated antigens (TAAs) were observed to be associated with ovarian cancer, including HER2/neu, P53, CA125, sTn, FR-α, mesothelin, NY-ESO-1 and CDR2 [40]. Although these antigens are commonly expressed in normal human cells, they may serve as potential targets for immunotherapy. The presence of tumor-infiltrating lymphocytes (TILs) was also positively correlated to the treatment outcome of patients with advanced ovarian cancer, as indicated by the increase in overall survival (OS) and progression-free survival (PFS) [41]. On the other hand, the presence of immunosuppressive regulatory T cells (T-regs), a subtype of T-cells that are important in the regulation of immune responses targeting self-antigens, was determined to be associated with a poor treatment outcome [42]. Such associations lay out the potential for immunotherapy as an alternative or complementary option to the current arsenal of ovarian cancer treatments (Figure 2). However, clinical trials (NCT02054806 and NCT02718417) engaging single-agent immune checkpoint inhibitors such as programmed death ligand 1 (PD-L1) inhibitors Pembrolizumab and Avelumab, in the treatment of recurrent and refractory ovarian cancers have only resulted in moderate success [42,43]. Noticeably, in the treatment of other malignancies, especially in solid tumors, chemotherapy exhibited the ability to elicit antigen expression, acting as a primer for tumor cells to become susceptible to the action of the mentioned PD-L1 inhibitors, improving the overall clinical outcome of treatment with immunotherapy [43]. Such findings have provided further optimism in treating ovarian cancer with immunotherapy, especially when combined with chemotherapy. 

However, cancer cell heterogeneity remains a challenge to the efficacy of such combined therapies. Tumor cells proliferate and survive based on the complex interaction between different pathways and molecular signalings. The targeting or inhibiting of one pathway may trigger other compensatory pathways, resulting in resistance to targeted therapy [44]. Tumors in a single patient may also respond differently to the same immunotherapy [45]. Cancer cells may show primary resistance in which the primary tumor does not respond to immunotherapy directly or acquired chemoresistance, where initial response to immunotherapy is observed, but disease progression upon carcinomatosis or relapse occurs after a period of treatment [46]. 

## 4. The Tumor Microenvironment (TME)-Stimulated Chemoresistance Is a Major Hurdle in the Successive Treatment of Ovarian Cancer 

While the different regimens of first-line chemotherapy may often improve the clinical response of patients, relapse of the disease is expected in more than 80% of patients with advanced ovarian cancer, with relapse frequently occurring within 2 years of the initial line of chemotherapy [38]. Out of these, more than half of the relapses have further developed chemoresistance, one of the most challenging problems to tackle in ovarian cancer treatment and management [33]. It has been proposed that the tumor microenvironment (TME) and intratumoral heterogeneity (ITH) serve as the major culprits in the development of chemoresistance in cancers [41]. For instance, the biological property of the ascites provides a region to accumulate malignant cellular and acellular elements, forming a unique TME that promotes the disease progression of ovarian cancer, modulating tumor cell behavior and resulting in tumor heterogeneity. The ascites consist of a heterogenous mixture of different cells, including malignant cells and stromal cells, with the latter playing a significant role in disease progression [47]. The stromal cells may be further classified as endothelial cells, mesothelial cells, adipocytes, fibroblasts, stem cells, immune cells, etc. Among them, the cancer-associated fibroblasts (CAFs), are directly involved in the promotion of proliferation and invasion of cancerous cells. Signal molecules released from CAFs are also engaged in promoting the development of chemoresistance in tumor cells [48]. 

Other than the multifaceted impact of the CAFs, metastatic cancer cells were also identified in the ascitic microenvironment. Ascites-derived cancer cells are hypothesized to play a key role in malignancy recurrence in ovarian cancer patients. These cells likely appear in the ascites in two forms: single cell form with adherent characteristics or an aggregate of non-adherent cancer cells known as spheroids. The non-adherent malignant cells that form the spheroids are often the invasive and metastatic sub-type of cells that originate from the primary tumor site and contribute to the relapse of the malignancy [49]. 

The acellular component, which is significantly influenced by the cellular composition of the ascitic microenvironment, has also been identified to play an important role in tumor progression due to the presence of a fluctuating mixture of pro-tumorigenic and tumor-suppressing factors. Several pro-tumorigenic cytokines were found to be elevated within the ascites, including IL-6, IL-8, IL-10 and VEGF. Among them, IL-6 is strongly associated with the response to therapy and overall prognosis in ovarian cancer [50]. Besides these, significant differences in the composition of oncometabolite like glucose, unsaturated fatty acids (UFAs), cholesterol and sphingomyelins in the malignant ascites of ovarian cancer have been confirmed through metabolomic profiling. The elevated level of glucose-1-phosphate (G1P), a metabolite in the process of glycogenesis, suggested that the rate of glucose metabolism within the ascites was also increased [51]. Furthermore, lipidomic analysis from our previous investigations showed that polyunsaturated fatty acids (PUFAs) were the main compositions of the ascites and ascites-derived ovarian cancer cells exhibited increased fatty acid desaturases activities [52,53], indicating that lipid metabolic activity of the metastatic ovarian cancer cells had been upregulated for tumor progression and aggression within the fatty acid-enriched ascitic TME. 

## 5. Augmented Fatty Acid Metabolism (FAM) Is Conducive to the Metastatic Aggression of Ovarian Cancer 

Peritoneal metastasis is the primary presentation of advanced-stage ovarian cancer and is defined as a trans-peritoneal invasion of the serosal linings of the peritoneal cavity by metastatic malignant cells from the primary tumor through a cascade of biological events [54]. Patients with peritoneal metastasis had a relatively abysmal prognosis compared to patients without carcinomatosis [55]. Within the peritoneal cavity, omental tissues are often the common site for metastasis arising from intraperitoneal tumors [56]. Most ovarian cancer cases (~80%) exhibited metastatic dissemination to the omentum accompanied by malignant ascites by the time of diagnosis [52,57,58]. Patients with malignant tumors on the omentum generally had a median survival time of about six months. Only 10–20% of patients survived beyond two years after surgical excision [59,60]. 

The omentum, a prominent peritoneal fold, has been regarded as the policeman of the abdomen based on its role in restraining inflammation and minimizing the spread of infections or local diseases in the abdominopelvic cavity. However, the latest studies have proposed that malignant ascitic/omental microenvironmental niches supply an abundance of soluble growth factors, proinflammatory cytokines and oncometabolite that are conducive to the progression and invasion of metastatic ovarian cancers [36,52,61]. In addition, the omentum is an organ with surplus fatty acids that are postulated to provide a vibrant energy source to feed the rapid tumor growth and promote homing and aggression of metastatic ovarian cancer cells [62]. Upregulated Warburg effect is a distinctive form of cancer cell metabolism with elevated levels of glucose uptake and increased conversion of glucose to lactose in the glycolytic pathway [63]. Nevertheless, we and others had just provided evidence that metastatic ovarian cancer cells could undergo metabolic rewiring to exploit upregulated lipid metabolic activities with elevated PUFAs content for tumor proliferation and progression in the fatty acid-enriched ascitic/omental TME [36,52]. Notably, our findings verified that aberrant expression of two major fatty acid desaturases, SCD1 and FADS2, accompanied by concomitantly enhanced activities of fatty acid desaturation and tumor aggressiveness, was observed in ovarian cancer cells derived from malignant ascites as compared with cancer cells from the primary ovarian tumor [53], indicating altered FAM is crucially associated with the oncogenic capacities of metastatic ovarian cancer cells. 

Recent reports also revealed that ovarian cancer cells cocultured with adipocytes, the significant cell population of omentum, resulted in the direct transfer of fatty acids from the latter to the former [57,64]. Besides, ovarian cancer cells cocultured with omental adipocytes induced adipocyte lipolysis and cancer cell β-oxidation, suggesting the lipid contents of adipocytes act as a vigor source for the malignant cells. Meanwhile, adipocytes have been shown to promote in vitro and in vivo tumor progression of ovarian cancer by providing energy to support cancer cell survival and aggressiveness during tumor colonization on the omentum [57]. Consistently, culturing ovarian cancer cells with ascites or omental conditioned medium (OCM) with plenty of free fatty acids by nature demonstrated a remarkable upsurge in cell growth, migration and invasion of metastatic ovarian cancer through upregulation of various signaling cascades like AKT/ERK/FOXM1 and TAK1/FASN/CPT1A/NF-κB axes [36,52,65,66]. Furthermore, it has been reported that the free fatty acids aid cancer cells in reducing oxidative stress by chaining mitochondrial oxidative metabolism, therefore forwarding metastatic dissemination [67], suggesting that various sub-clones of the metastatic ovarian cancer cells should have evolved compensatory signaling pathways against sensitivity to lipid peroxidation upon remodeling for enhanced lipid metabolic activity. Thus, the rephrasing of lipid metabolism in metastatic ovarian cancer involves the modulation by a sophisticated and coordinated network of signalings [68], and targeting the altered FAM may act as a potential remedy to sensitize subsets of metastatic ovarian cancer cells in the fatty acid-enriched peritoneal ascitic/omental TME. 

## 6. Targeting Aberrant FAM with Ferroptosis-Inducing Compounds (FINs) as the Swiss Army Knife against Metastatic Ovarian Cancer 

Given that metastatic ovarian tumors experience a metabolic adaptation from glycolysis to fatty acid β-oxidation to generate ATP for supporting the high energy requirement of the fast-growing cancer cells in the free fatty acids-enriched ascitic/omental microenvironment [69]. Among all the risk factors involved in enhanced lipid metabolic activities, the upregulated reactive oxygen species (ROS) is significantly related to the dysregulated lipid metabolism. ROS plays an essential role in cell signaling and tissue homeostasis [70]. However, during persistent oxidative stress, ROS not only can oxidatively modify proteins and initiate DNA damage, but they also can cause an alternation of membrane permeability and induce lipid peroxidation by directly reacting with the PUFAs-rich membrane phospholipids, leading to different types of programmed cell death (PCD) such as apoptosis and ferroptosis [71,72,73]. Thus, it is believed that well-evolved and sophisticated antioxidant systems are present to protect metastatic ovarian cancer cells from ROS-induced lipid peroxidation chain reactions. 

**Figure 3:**
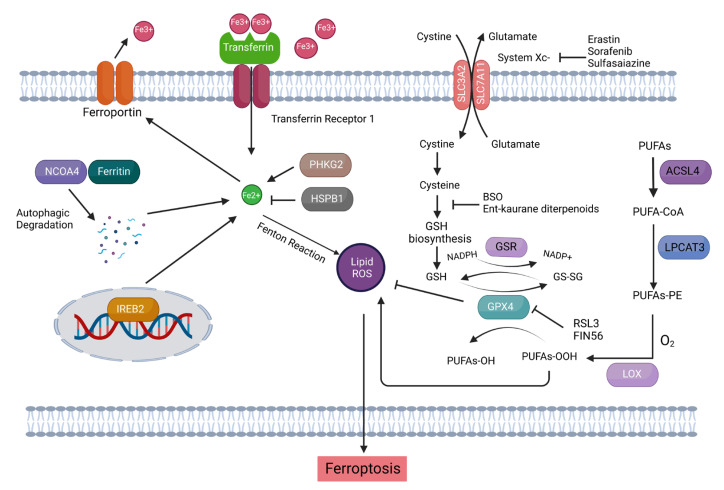
The mechanisms of ferroptosis. Ferroptosis is induced through inhibition of system Xc- or Glutathione Peroxidase 4 (GPX4) activity, ultimately resulting in cell death. System Xc-, a cystine/glutamate antiporter, transports intracellular glutamate to the extracellular space in exchange for the extracellular cystine into the cell, which is subsequently transformed into cysteine (Cys2), a reduced form of cystine, for the synthesis of glutathione (GSH). Exploiting GSH as the cofactor, GPX4 exerts its phospholipid peroxidase activity to prevent the accumulation of lipid reactive oxygen species (ROS) within the cell by reducing the endogenous lipid peroxides from PUFAs-OOH to PUFAs-OH. Therefore, the small molecule inhibitors, such as Erastin and Sorafenib, -mediated inhibition of system Xc- causes depletion of GSH and inactivates GPX4 that eventually leading to the accumulation of lethal lipid peroxides and the initiation of ferroptosis. In addition, direct inhibition of GPX4 through inhibiting its activity, e.g., ferroptosis inducer (1S, 3R)-RSL3, or promoting its degradation, e.g., genetic knockout of GPX4 is another way of ferroptosis execution. Besides lipid peroxidation, the excessive number of irons is also an essential criterion for ferroptosis induction. Circulated iron was combined with transferrin (TF), an iron-binding protein, in the form of ferric ions (Fe^3+^) and transported into cells by TFR1-mediated endocytosis. Ferric ions were deoxidized to ferrous ions (Fe^2+^) by iron oxide reductase STEAP3, which then are capable of catalyzing lipid peroxides to form damaging free radicals via Fenton Chemistry. When these lipid peroxides cannot be eliminated in time within the cell, ferroptosis may occur [93].

Ferroptosis is a novel means of PCD, and its term and features were firstly coined and described by Brent R STOCKWELL and Scott J DIXON, not until 2012 [74] (Figure 3). Given the key features of ferroptosis that it is characterized by an enormous accumulation of iron and lipid peroxides during the cell death process, ferroptosis is biochemically distinct from other forms of PCD such as apoptosis, anoikis and autophagy [75,76]. Besides as an iron-dependent PCD, ferroptosis is also a ROS-reliant cell death with main morphological changes such as loss of cell membrane integrity, partial release of cell contents, chromatin condensation, fracture of mitochondrial membrane, reduction in mitochondrial volume, marked loss of mitochondrial cristae, etc. [77,78,79,80,81], arising from the incisive membrane lipid peroxidation induced by the oxidative stress [82]. To this end, elevated lipid metabolism is intimately correlated with cell vulnerability to ferroptosis [75]. Nowadays, the distinguishing role of ferroptosis in repressing malignant cells is gradually being identified in biomedical research and has the potential to be further developed into a new type of anticancer remedy known as ferroptosis-initiating therapies (FITs) [83]. For instance, a wide variety of ferroptosis-inducing compounds (FINs), including pharmaceuticals like Erastin and Sulfasalazine and natural compounds like Curcumin analog, have been reported to mitigate tumor cell growth and overcome resistances toward chemotherapy and targeted therapy in some malignancies such as lymphoma, lung carcinoma, renal cell carcinoma, glioma, breast cancer, etc. through ferroptosis induction [53,84,85,86,87]. The synergistic effect of anticancer therapies in combination with ferroptosis inducers in ovarian cancer was also observed in some independent studies (Table 1). Indeed, our latest publication revealed that MAP30, one of the bioactive constituents in bitter melon extract (BME), decreased ATP production in OCM-cocultured ovarian cancer cells and reduced the GSH/GSSG ratio and GPX4 expression similar to Erastin in a dose-dependent manner, which inversely correlated with the escalated cellular ROS and lipid peroxidation levels in ovarian cancer cells through AMPK signaling [36]. More importantly, we have novelistically demonstrated that pharmaceutical inhibition and genetic ablation of SCD1/FADS2 fatty acid desaturases in ascites-derived metastatic ovarian cancer cells not only disrupted the GSH-GPX4 system and enhanced iron-mediated lipid peroxidation that led to ferroptotic cell death but also synergized the anticancer effect of Cisplatin upon concurrent treatment of Erastin to suppress peritoneal metastasis of ovarian cancer [9,53], denoting the potential combinations of FINs and FAM modulators as a novel means of therapeutic regimens for tackling chemoresistance and carcinosis of ovarian cancer. Thus far, this is an innovative report about the inhibition of FAM that could trigger FINs-induced ferroptosis in ovarian cancer, at least in part, by modulating SCD1/FADS2. Accordingly, a series of enzymes, including ACSL4 and LPCAT3, involved in the regulation of lipid metabolism can potentially possess the sensitivity of ovarian cancer cells to ferroptosis [88,89]. Besides ACSL4/LPCAT3, other lipid metabolic enzymes such as acetyl-CoA carboxylase (ACAC), which mediates fatty acid synthesis, and the lipoxygenase (LOX) family, which promotes the peroxidation of arachidonic acid (AA) and adrenergic acid (Ada), can elevate lipid peroxidation and subsequent oxidative damage to the cell membranes [90,91,92]. Hence, these associated molecular targets/pathways can be potentially explored and targeted to induce ferroptosis during FITs for erasing peritoneal metastasis of ovarian cancer. 

Another proposition for boosting the anticancer efficacy of ferroptosis is iron modulation [99]. Iron addiction is a discrete characteristic of many malignant cells, and they develop various mechanisms to guarantee the iron supply for cell proliferation and aggression by concurrently accumulating iron and preventing iron efflux [9,53,100,101,102]. For example, the systematic iron pool in leukemia patients had risen [103], and the expression level of transferrin receptor (TFR), an iron transport protein, was significantly higher in leukemia cells than in normal cells. The treatment of antitumor drugs together with TRF targeting approaches, remarkably reversed the resistance of leukemia cells to Doxorubicin and Verapamil [104,105]. Consistently, tumor-associated macrophages (TAMs), particularly the M2 phenotype, in the TME have been reported to act as a source of iron for promoting tumor growth in an iron-dependent manner. Not only a substantial iron reservoir was observed in the inflammatory stromal cells from the primary tumors and axillary lymph nodes of breast cancer, but the malignant cells also exhibited an iron acquisition characteristic by overexpressing hepcidin and transferrin receptor 1 (TFR1) and concomitantly downregulating ferritin, whereas the TAMs within the TME manifested an iron delivery feature by expressing ferroportin and ferritin [106]. Furthermore, considering the iron-overdosed properties around the cancerous neoplasm [9], serous subtype ovarian cancer cells were more susceptible to lipid peroxidation and Fenton reaction due to the upregulated TFR1-mediated iron uptake [98]. A similar phenomenon existed in ovarian tumor-initiated cells (TICs) that they had higher expression of TFR1 and lower level of ferroportin-1 (FPN) and exhibited increased sensitivity to Erastin [102], hinting that combo of FITs and FAM targeting strategy would be more effective in selective subtypes of ovarian cancer. On the other hand, the involvement of iron in lipid peroxidation enzymes such as LOX and cytochrome P450 oxidoreductase (POR) provides us further insight into how iron plays a significant role between the lipid metabolic process and ferroptosis. These lipid peroxidation enzymes require iron as a cofactor to carry out their enzymatic activity in facilitating phospholipid peroxidation [107,108]. Likewise, the aforementioned fatty acid desaturases, SCD1 and FADS2, are also iron-containing enzymes that the combined SCD1/FADS2 need iron binding at the center of their catalytic domain to execute enzymatic activities [53,109,110]. That is why our previous study on the inhibition of SCD1/FADS2 could lower the iron-binding capacity and induce the upsurge of the cellular labile iron pool, which in turn, led to deregulated ROS deposit and lipid peroxidation in ovarian cancer cells [53], suggesting novel combination regimens based on FITs would be helpful to improve treatment outcomes. 

## 7. Conclusions and Perspectives 

Among the fatal gynecological malignancies in female populations, ovarian cancer is one of the most notorious. Although tumor marker detection had been commonly exploited in the clinical management of cancer to assist the screening, diagnosis, prognosis and recurrence prediction as well as post-treatment monitoring [111], the lack of reliable biomarkers and the subsequent delayed detection of the disorder conferred ovarian cancer the silent killer [66]. Peritoneal metastasis is prognostically relevant in patients with advanced ovarian cancer and is the major cause of cancer-related mortality [66,112]. The peritoneal TME, especially the fatty acids-enriched ascites and omental surfaces, is a multiplex milieu that exerts significant impacts on malignant metabolic adaptation, promoting tumor development, metastatic progression and aggressive tumor relapse after frontline treatments. Persistent micrometastasis is clinically undetectable and represents the spore that highly contributed to disease recurrence. Developing unprecedented capabilities for identifying and understanding specific molecular alterations as novel MRD targets to transform the care for women with ovarian cancer has been a research focus. Therapeutic strategies for exploiting pharmaceuticals to trigger PCDs in tumor cells during cancer treatment have been investigated for a long time. However, evasion of the cell death process, especially apoptosis due to intrinsic and acquired resistance, is a hallmark trait of cancer, bringing about a significant obstacle to hitting the targets in disease treatment. Recent findings discovered that malignant cells eluding from other regulated forms of cell death, including apoptosis and autophagy during tumor progression, remained sensitive to ferroptosis [9,93,113]. In addition, malignant mesenchymal cells, which are resistant to various treatments and are prone to aggressive metastasis, are highly susceptible to ferroptosis [114], inferring that initiating ferroptosis could be a practical therapeutic approach for anticancer therapy. Since excessively high FAM supports metastatic growth and accelerates the oncogenic capacities of advanced ovarian cancer cells in the fatty acids-enriched microenvironment of the peritoneal cavity, it is conceivable that the overwhelming buildup of iron-dependent lipid peroxides is regarded as the decisive executioner eliciting ferroptosis cancer cell death when the endogenous oxidative stress defense abilities are being flooded. Ferroptosis induction may therefore give rise to a distinctive potential to at least obliterate several sub-clones of metastatic ovarian cancer cells. Yet, there appears to be quite a long way to go before the translational application. Although there is emerging evidence displaying the anticancer effect of ferroptosis initiation in numerous experimental models, there is a paucity of clinical trials about ferroptosis-associated agents showing tumor shrinkage or prolongation in PFS [115], implying that sole treatment of ferroptosis inducers in practice may not be as effective as they are in in vitro studies and alternative combination therapy with FAM modulation may complement each other to fight against malignant metastasis of ovarian cancers. 

Instances of the combined effects of ferroptosis initiation as a result of targeting strategies on the signaling pathways involved in lipid metabolism have been documented. For example, the upregulation of ACSL4 and TFR1 was attributed to the ability of the YAP-TEAD complex to promote ferroptosis in mesothelioma [114,116]. On the other hand, AMPK triggered ferroptosis initiation by blocking system X_C_^−^ activity [116,117,118,119]. Similar synergizing outcomes upon the combination of standard therapeutics such as radiotherapy, chemotherapy and immune checkpoint blockade (ICB) with drugs and compounds involved in ferroptosis function to promote cancer cell death have also been described [116,120,121,122]. Our aforesaid experiments consistently demonstrated that inhibition of fatty acid desaturases equipoised redox-driven ferroptosis in ascites-derived ovarian cancer cells with high demand on lipid metabolic activity and sensitized the metastatic ovarian cancer cells to Cisplatin-induced cell death, additionally strengthening the link between FAM and ferroptosis and supporting the idea of modulating ferroptosis through FAM manipulation together with other potential treatments to achieve favorable interventions for metastatic ovarian cancer. 

On the other hand, mounting evidence has proposed that metastatic traits are significantly attributed to epigenetic alternations, which augment tumor oncogenic properties during malignant progression [66,123]. In fact, TME plays a vital role in shaping the aberrant epigenetic mechanisms in cancer metastasis that includes transcriptional control of microRNAs (miRNAs) via specific DNA hypermethylation on tumor suppressor gene promoters [66,124]. Interestingly, miRNAs and long non-coding RNA (lncRNA) are increasingly recognized as critical mediators in the regulation of ferroptosis [83]. For example, miRNA-27a [125] and miR-26b [126] can respectively modulate ferroptosis induction in bladder cancer and breast cancer cells by targeting the cystine/glutamate exchanger SLC7A11, while lncRNA P53RRA can promote ferroptosis via nuclear sequestration of P53 [127]. Furthermore, miRNAs like miR-210 [128] and miR-20a [129] are involved in the regulation of iron metabolism-related genes such as *TFR1* and *FPN*, respectively, that are important for iron uptake/export. Besides these, miR-144–3p [130], miR-224–5p [131] and miR-205 [132] can reduce the expression of the lipid metabolic genes such as *ACSL4* and *LPCAT3*, suggesting that miRNAs may mitigate ferroptosis by targeting potential executioners of ferroptotic cell death. Similarly, miR-33b is known to regulate FAM and target lipid metabolism genes. Our earlier report showed that restoration of epigenetic silenced miR-33b in OCM-cocultured ovarian cancer cells could remarkably impair cell proliferation, metastasis, OCM-upregulated lipid metabolic activities and ATP production by reducing the expression of FASN and CPT1A [66], suggesting a conceivable prospective, yet to be demonstrated that, modulating ferroptosis via targeting the FAM associated miRNAs as a molecular therapeutic choice for impeding ovarian cancer metastases. Thus, a deeper insight into the molecular mechanisms involved between ferroptosis tolerance and altered FAM in metastatic ovarian cancer will contribute to this goal. 

## Figures and Tables

**Table 1:** Summary of studies on ferroptosis inducers in ovarian cancer. The anticancer effects of the chemotherapeutic or targeted therapeutic drugs are enhanced upon combination with ferroptosis inducers.

Combination Drugs	Target	Mechanism	References
Erastin & Cisplatin	System Xc-	Erastin inhibited system Xc- and enhanced the cytotoxic effects of Cisplatin to eradicate tumor cells.	[94]
Erastin & Docetaxel	SLC7A11	Combination of Erastin and Docetaxel significantly promoted cell apoptosis, and induced cell cycle arrest at G2/M in ovarian cancer cells with ABCB1 overexpression.	[95]
Sotuletinib (BLZ945) & Docetaxel	CD8^+^ T cells	BLZ945 combined with Docetaxel increased the infiltration of CD8^+^ T cells in tumor tissues.	[96]
SCD1 inhibitors & RSL3/Erastin	Lipid peroxidation ROS	Stearoyl-CoA desaturase 1 (SCD1) inhibitors reduced an endogenous membrane antioxidant, CoQ_10_, which significantly potentiated the antitumor effect of Erastin and RSL3 in ovarian cancer cells.	[97,98]
